# NIMBYism and Strategies for Coping with Managing Protests during the Establishment of Community Mental Health Facilities in Taiwan: Insights from Frontline Healthcare Professionals

**DOI:** 10.3390/healthcare12040484

**Published:** 2024-02-17

**Authors:** Ching-Teng Yao, Hong Hong, Chien-Hsun Li

**Affiliations:** 1 Master Program of Long-Term Care in Aging, Kaohsiung Medical University, Kaohsiung 80708, Taiwan; 2 Bachelor Program of Senior Health Promotion and Care Management for Indigenous People, National Changhua University of Education, Changhua 50007, Taiwan; oscarhong@cc.ncue.edu.tw; 3 Integrated Center of Healthy and Long-Term Care, Kaohsiung Municipal Siaogang Hospital, Kaohsiung Medical University, Kaohsiung 80708, Taiwan; gavli@kmu.edu.tw

**Keywords:** community mental health facility, frontline healthcare professional, not-in-my-backyard, resistance response strategies, qualitative analysis

## Abstract

Taiwanese community mental health facilities encounter opposition/resistance from residents, commonly known as “Not-In-My-Backyard” (NIMBYism). This study investigated NIMBYism during the establishment of such facilities and how they respond to such resistance. A qualitative study through semi-structured interviews was used to obtain purposively sampled data. Fifteen frontline healthcare professionals from community mental health facilities in Taiwan were interviewed individually, using an organizational analysis structure. Data were analyzed using qualitative content analysis. Two themes: “Reasons for Resident Resistance” and “Institutional Response Strategies”, two categories, and 11 subcategories emerged. The findings demonstrated the following: (1) Reasons behind residents’ resistance toward establishing community mental health facilities are diverse. (2) Communities lack understanding regarding people with mental disorders, leading to irrational beliefs. (3) Fear and negative perceptions toward people with mental disorders exist. (4) Strategies employed by the facilities include providing community services to foster amicable relationships, organizing community outreaches, training people with mental disorders within communities, nurturing neighborhood connections, establishing and sustaining friendships within communities, inviting residents to visit community mental health facilities or introducing the facilities to communities, and leveraging governmental support. The government should adopt regulations or laws to reduce discrimination, promote human rights, and legislate to demarcate the use of community land.

## 1. Introduction

Over the past two decades, there has been an almost 155-fold increase globally in disability-adjusted life years (DALYs) attributed to mental disorders, particularly noticeable within high-income countries [1]. Mental disorders have emerged as the third leading cause of the global disease burden, following closely behind cardiovascular diseases and cancer [2]. In Taiwan, the number of people seeking treatment for mental disorders has steadily risen over the past decade, escalating from 1.52 million people in 2011 to 2.058 million in 2020. However, over 70% of people with mental disorders do not seek any professional assistance [3]. In 2016, a random killing took place in Taipei’s Neihu district, in which a four-year-old girl was killed in the presence of her mother. Due to several recent incidents in Taiwan where people with mental disorders caused harm in communities, the Taiwanese government announced the initiation of the Second Phase of the Social Safety Program in 2021. This initiative aims to establish a community-based mental healthcare system specifically tailored for people with mental disorders [4]. However, the establishment and promotion of these community mental health facilities have faced significant opposition from community residents. In addition to expressing concerns about the perceived lack of safety associated with locating these facilities within communities, residents harbor the belief that people grappling with mental disorders often exhibit inclinations toward violence and could potentially incite societal crime [5]. The public’s misconceptions about mental illnesses result in the emergence of prejudice, subsequently leading to discrimination. Consequently, numerous challenges have arisen in the advancement of many community mental health facilities. Taiwanese attitudes toward people with mental disorders have remained deeply entrenched and unchanged over the past few decades [6].

Regarding the establishment of mental health facilities within communities, the public often holds stigmatizing and discriminatory attitudes [7,8]. Structural discrimination is evidenced by adverse attitudes, exemplified by opposition to the allocation of financial resources for the care of people experiencing mental health issues and resistance toward the provision of mental health rehabilitation services within communities [9]. The aforementioned acts of stigmatization and discrimination exert detrimental effects on the recovery trajectory of people coping with mental disorders, simultaneously fostering a negative impact on their self-perception. Moreover, these adverse effects, particularly the stigma associated with mental disorders, subsequently impede the advancement of mental health policies and the implementation of relevant service measures [10,11]. Hence, to mitigate these adverse effects, both the community and public policies play pivotal roles in facilitating the recovery journey of people grappling with mental disorders [12].

In light of current global mental health development trends, there has been a shift in the approach to mental disorders from the previous emphasis on institutionalization and long-term hospitalization toward a community-oriented mental health model [13]. The most ideal environment for the recovery of people with mental disorders is one that is familiar to them and minimally restrictive, aligning with the communities where these individuals reside and to which they are accustomed. Hence, countries worldwide have been progressively closing psychiatric institutions, making the community-based mental health model the mainstream approach to advancing mental health policies. The United Nations Convention on the Rights of Persons with Disabilities (CRPD) underscores the equal rights of people with physical and mental disorders to live in the community on par with others. To ensure this parity, it advocates the implementation of effective and appropriate measures that enable people with physical and mental disorders to fully exercise these rights. The focus is specifically on facilitating those individuals to live a life of dignity and equality alongside others [14]. In Taiwan, the Mental Health Act has been established to protect the rights of people with mental disorders concerning their care and treatment. Article 1 of the law explicitly states the objective to support and assist people with mental disorders in living within the community. It aims to aid these individuals in gradually adapting to social life by providing community mental health (rehabilitation) services that focus on enhancing their psychological, social, and daily life skills [3].

The “Not-in-my-back-yard” phenomenon (NIMBYism) encompasses the emotional, attitudinal, cognitive, and behavioral opposition of individuals or communities against the use of a particular land or buildings [15]. While experts or technical bureaucrats may assert the absence of technical issues regarding these development projects or land use methods, they often encounter robust opposition or resistance/protests from residents. In Taiwan, starting from the first protest against the establishment of a rehabilitation center for children with physical and mental disorders in 1983, a series of community protests against the integration of facilities for people with physical and mental disorders into local neighborhoods has persisted. This opposition has been consistent, especially regarding the establishment of mental health facilities within communities [16]. These incidents highlight that mental health (rehabilitation) facilities have become NIMBY facilities, reflecting the deep-seated apprehension and concerns of community residents regarding mental disorders. People with mental disorders bear the brunt of stigmatization and exclusion within communities. As Taiwanese society continues to stigmatize and exclude/marginalize mental disorders and community mental health facilities, there is a pressing need for in-depth exploration into experiences concerning the establishment of community mental health facilities with the aim of fostering the integration of people with mental disorders into the community. This study focused on frontline healthcare professionals involved in the establishment of community mental health facilities for people with mental disorders within communities. Through interviews, the study aimed to comprehend these healthcare professionals’ experiences in interacting with the public during protest incidents, the measures taken at the time, and the underlying thought processes. Engaging with existing literature on NIMBYism, the study sought to offer fresh perspectives on current relevant research while deriving concrete recommendations from real-life experiences. These insights aimed to provide valuable guidance for future policy revisions or service measures in the mental health domain.

## 2. Methods

This study adopted a qualitative research approach, emphasizing participants’ descriptions and understanding of contextual circumstances. Employing in-depth interview techniques, the study focused on frontline healthcare professionals directly engaged in managing protest incidents involving community mental health facilities. These professionals, acting as representatives of the facility, were involved in on-site communication and coordination with protesting residents, government officials, and representatives throughout the process of handling/managing these protests. This study investigated how frontline healthcare professionals navigate community resistance and opposition when establishing community mental health facilities. It explored the strategies employed by these professionals to ensure the smooth establishment of these facilities despite community pushback. These strategies encompassed communication and persuasion tactics tailored to address public opposition/resistance, with the goal of allaying community residents’ concerns. Additionally, it examined how healthcare professionals engage in dialogue and provide social education to integrate people with mental disorders into the community.

### 2.1. Participants

The selection of research participants primarily utilized purposive sampling. First, the researchers identified representative entities involved in protest incidents concerning community mental health facilities based on their comprehension of such events. Second, through referrals and suggestions from interviewees, a snowball sampling method was employed to include additional entities that could enrich the diversity and richness of the research data. This approach allowed the interviews in this study to encompass various service facilities and types of protest incidents across different regions. Third, frontline healthcare professionals actively engaged in managing protest incidents were specifically selected as the participants, each possessing a minimum of one year of experience in their respective positions. Moreover, their full commitment to engaging in comprehensive in-depth interviews was a prerequisite for their selection. The researchers explained the study’s objectives to each participant and ensured compliance with academic ethical standards, obtaining signed consent forms from all participants. The study was conducted from March 2023 to October 2023. Fifteen frontline healthcare professionals from community mental health facilities were interviewed for this study.

### 2.2. Research Ethics

This study was approved by the Institutional Review Board and was conducted with the consent of the institution. The researchers explained the objective and method of this study to the participants and acquired their written consent. Their anonymity and confidentiality were strictly protected. All research data were encoded to ensure the anonymity of the participants and used only for academic research purposes. The participants were permitted to withdraw from a session or quit the study altogether during the research procedure for any reason.

### 2.3. Data Collection

This study primarily utilized semi-structured, in-depth interviews. Prior to the interviews, an interview outline was sent to the research participants via email. The interview outline encompassed several sections, including the demands expressed by community residents during protests, the attitudes of government agencies, strategies employed by facilities to resolve protest incidents and their coping mechanisms, and approaches involving dialogues with community residents and social education. The selection of interview locations and timing prioritized the considerations of the interviewees. The interviews, lasting between 60 and 90 min, were predominantly conducted at the participants’ current service entities, with a full recording conducted upon their agreement. Each research participant underwent two face-to-face interviews. In addition to the in-depth interviews, participants proactively provided various relevant documents preserved by the institution during the events, such as meeting records from negotiation processes, official correspondences, legal opinions, and other related materials. These documents served as crucial background information to comprehend the interview content and were used for cross-referencing during data analysis.

### 2.4. Data Analysis

The qualitative content analysis method proposed by Graneheim and Lundman was employed to analyze the collected data in this study [17]. The study followed four steps: (1) Multiple readings of the verbatim transcripts were conducted to gain an understanding of the overall content; (2) Textual data were read, coded, and continuously compared and contrasted to comprehend the underlying meanings and relationships; (3) Inductive analysis of the data was performed to identify common themes, categorizing data with similar meanings to develop core and sub-categories; (4) The study outcomes were derived by analyzing the meaning, patterns, and concepts within the gathered data content.

### 2.5. Rigor

We examined the rigor and trustworthiness of this study based on the following four criteria proposed by Guba and Lincoln on the precision of qualitative research [18]: (1) Credibility: The researchers had extensive experience in studying community-based mental health policies and were well acquainted with the process of promoting mental health policies in Taiwan. Furthermore, the researchers had received comprehensive training in qualitative research, demonstrating practical proficiency in conducting interviews and performing qualitative analysis. In addition, regular discussions with qualitative research experts were an integral part of the research process. (2) Transferability: Interviews were accurately and truthfully transcribed verbatim for presentation in this study. Transcriptions of the interview content were returned to participants for correction. (3) Dependability: We invited two community mental health professionals with broad experience in qualitative research to review and modify the classification of the findings. (4) Conformability: The researchers safeguarded all the reflective field notes and records of data analysis in this study for future verification and reference. At the final stage of the study, the participants were given the opportunity to review and confirm the research outcomes.

## 3. Results

In this study, 15 frontline healthcare professionals from community mental health facilities involved in handling/managing protest incidents were interviewed. The average job tenure of the participants was 6.8 years. The majority of the participants had a background in social work, followed by nursing. Most participants had professional experience ranging from seven to nine years. Regarding the organizational attributes, privately established institutions were predominant, and in terms of geographical distribution, the majority were located in southern Taiwan (Table 1).

Following the analysis of the interview data, the findings are structured into two major sections for presentation. The first part outlines the reasons behind community members’ resistance, which were categorized into four distinct categories. The second part delineates the strategies adopted by institutions in response to resistance from community residents, classified into seven distinct categories (Table 2).

### 3.1. Reasons behind Community Residents’ Resistance against Community Mental Health Facilities

#### 3.1.1. Diverse and Varied Negative Perceptions

The reasons given by the residents for their resistance against community mental health facilities were multifaceted, differing from concerns associated with pollutant facilities. These reasons encompassed a wide spectrum, ranging from landscape disruption, feng shui disturbances, impacts on air or water quality, effects on child development, and concerns about personal safety, to potential decrease in real estate value.

*All the instigators are in a single stance… The reasons they give are essentially the same, like how we will impact their safety, cause disturbances, or decrease property values. These reasons are almost always uniform.*

(M1)

*They used to say things like, “My child will pick up bad habits”, “My child will become less intelligent”, and then they would mention behaviors like spitting or making noise and so on. Then, “My son is about to get married.” That’s exactly what they said, along with the idea that his wife wouldn’t move in. Those were the opinions we heard at that time.*

(M14)

*I mentioned taking them (people with mental disorders) to the park. But then they said, “No way! Taking them to the park is risky. These people with mental disorders are just too dangerous! I have kids around; they might catch mental disorders from the air.” It’s a lot of unfounded imagination like this!*

(M7)

*They also started fearing our behavior and words!… Worried not only for themselves but also for their families, particularly the children, who might feel anxious or scared! This scenario brings about psychological stress for them…*

(M3)

#### 3.1.2. Social Stigmatization and Exclusion, Attributing Unrelated Community Matters to People with Mental Disorders

Due to societal stigmatization and exclusion in terms of mental disorders, many residents tend to attribute unrelated community matters to people with mental disorders.

*When disturbances happen in the community, like someone getting intoxicated or creating a ruckus outside, they tend to attribute it all to people with mental disorders! But in reality, these incidents aren’t caused by those individuals. There’s a habit among other residents to link all these negative occurrences in the community solely to the presence of people with mental disorders.*

(M12)

*They always pin the blame on people with mental disorders, but it’s not true! Even when the elevator is out of order, they claim it’s the fault of those with mental disorders. When someone defecates or urinates inside the elevator, they attribute it to people with mental disorders! They claim, “Mentally ill people do these things everywhere”, and they place blame for various negative community behaviors on those with mental disorders. But later, it’s confirmed that it wasn’t caused by them.*

(M9)

#### 3.1.3. Fear of People with Mental Disorders: Reflection of Social Values

Community residents also acknowledge that their fear of people with mental disorders reflects the collective societal value system. The current intense opposition from residents only arises because the institution’s establishment happens to be located in a specific community.

*Some residents mention that “I previously cherished the tranquility of my home. However, with the constant flow of people entering and leaving the mental health facility, things have become complicated. Each day, various patients with mental disorders visit our building, creating a sense of insecurity in our community. These individuals are like unpredictable time bombs.”*

(M2)

*The media often reports incidents of people with mental disorders attacking others, portraying them as having violent tendencies. So, when they are set to come to our community, residents express deep fear, feeling that the community will become unsafe.*

(M6)

#### 3.1.4. Misunderstanding of People with Mental Disorders: Riddled with Irrational Beliefs

Some residents may not directly express their concerns, or they may not fully understand the underlying reasons for their deep-seated fears. Hence, they tend to voice various objections and doubts. Some of these doubts can be clarified or rebutted with concrete evidence, making them relatively easy to verify. Nonetheless, a substantial portion of these doubts prove challenging to persuade or disprove.

*Some residents mention that home is their peaceful haven. However, after the establishment of such a facility, the people with mental disorders who visit are like a ticking/unpredictable time bomb. The once tranquil living environment will become unsafe and filled with anxiety throughout the community.*

(M8)

*Some residents mention that “people with mental disorders might potentially pose a threat, and by having such a facility in our community, it puts the elderly and children at risk. We have to be extremely cautious when leaving our homes, fearing for potential attacks within the community.”*

(M11)

### 3.2. Strategies Employed by Institutions in Response to Community Residents’ Resistance

Based on the experiences of research participants, there are seven categories of communication strategies adopted by community mental health facilities in response to resistance from community residents. These strategies include providing community services, organizing or participating in community outreach activities, offering independent living training for people with mental disorders, facilitating the establishment and sustenance of friendships, nurturing local neighborhood connections, facilitating the employment of people with mental disorders within the community, and inviting community members into the facility or introducing the facility to the community.

#### 3.2.1. Providing Community Services to Foster Amicable Relations

All research participants highlighted that community service is an effective method to establish positive relationships with community residents, promoting integration into the community. These methods include: (1) providing community cleaning services; (2) participating in community recycling activities; (3) caring for the disadvantaged within the community; and (4) assisting with agricultural tasks in the community.

*We visited a care center where approximately a dozen wheelchairs were available at once. The residents utilizing these wheelchairs routinely visit the community park every Friday for sun exposure, and our involvement includes assisting them by individually pushing the wheelchairs. Each resident is responsible for pushing one wheelchair.*

(M2)

*The majority of residents here are involved in agricultural work. With the population outflow, many elderly people are still actively engaged in farming despite their advanced age. However, there is a shortage of laborers. When our facility lends a hand, there are usually around 20 to 30 additional laborers. The land we work on is quite extensive, covering several acres. With our sizable workforce, we quickly manage tasks like weeding, benefiting from our efficiency due to the larger number of hands.*

(M5)

#### 3.2.2. Organizing or Engaging in Community Outreach Activities

These include involving people with mental disorders in large-scale community events organized by the community, community education programs commissioned by local governments, or actively conducting mental health seminar sessions.

*As community events are open to the public and accessible to everyone, activities such as performances and competitions include both people with mental disorders and the general public. The aim is to enable them to engage together, participate in competitions, and play alongside each other, with the hope of fostering a genuine understanding of people with mental disorders.*

(M5)

*Every year, there is a sports event in the community, and we always participate. We are part of a very popular and energetic cheerleading team on the field. We rush around, shouting as loudly as we can each time. It’s our way of getting the community residents to understand us as much as possible.*

(M7)

#### 3.2.3. Providing Independent Living Training for People with Mental Disorders in the Community

The independent living training for people with mental disorders in the community serves as a significant means to foster understanding among community residents regarding those individuals. This training involves a focus on attire and personal hygiene to prevent the potential social exclusion of people with mental disorders within the community due to any perceived unconventional appearance. Furthermore, it provides training in interpersonal communication, expression, and transportation skills to those individuals.

*They have great independent living skills. So, they are likely to immediately blend in with others when going out… I wanted their attire and appearance to be tidy and their hair to be well-groomed. So, my primary focus is good personal hygiene.*

(M8)

*If I notice any inappropriate remarks or behavior while they (people with mental disorders) are interacting with others, I wait for them to come in and then have a discussion. I talk to them about those behaviors and advise them on what to be careful about when communicating with other residents in the community…*

(M12)

#### 3.2.4. Nurturing Local Neighborhood Connections

The research participants shared instances where their community mental health facilities faced protests either before or during establishment. When a facility has faced or is currently facing community protests, people with mental disorders receiving services there often feel the pressure of being excluded by the community. Thus, establishing and maintaining positive relationships with the neighborhood becomes a crucial factor for these institutions to facilitate the integration of people with mental disorders into the community.

*Those with whom we have more interaction could be, for example, the owners of buffet restaurants or convenience stores … Similar types of connections tend to foster mutual interactions. From a community perspective, this situation could be perceived as a mutually beneficial relationship.*

(M11)

*Actually, in this aspect, we’ve put in quite a bit of effort. We actively encourage them (people with mental disorders) to say hello, remember people’s last names, and greet them with phrases like “Good afternoon, Mrs. So-and-so” when they see them. As they greet others over time, connections are built. Gradually, other residents nod back in response. Eventually, they might ask about people with mental conditions, like “What’s he like?” and we’ll clarify and explain at an appropriate…*

(M5)

#### 3.2.5. Facilitating the Establishment and Sustenance of Community-Based Friendships

The course of an illness frequently leads people to undergo diverse forms of loss. Especially, when an individual requires continuous medical or social care, it could entail confronting physical or cognitive impediments/impairments or experiencing relocation and separation from significant others due to caregiving demands. These circumstances might also disrupt ties with close friends and family, contributing to the individual enduring persistent feelings of isolation and melancholy. Therefore, facilitating the establishment and sustenance of friendships between people with mental disorders and other residents in the community is one of the crucial ways to facilitate the integration of those with mental disorders into the community.

*Encouraging them to interact with other community residents is something I feel like we’re doing all the time, every day!… In reality, people with mental disorders are still a minority in the community! So, whenever they go out, they naturally encounter other community residents.*

(M14)

*So, we encourage them to step out of the community mental health facility and establish more enduring relationships with residents… Maybe they just want someone to talk to, or perhaps they want to learn something. We try our best to encourage them to participate in various activities, not necessarily limited to those exclusively for groups with mental disorders.*

(M10)

#### 3.2.6. Inviting Residents to Visit the Community Mental Health Facility or Introducing the Facility to the Community

*Our friends with mental disorders are just like us! I want them to enjoy the same comfortable living in the community as I do. Hence, we invite community residents to get to know our facility and help them gain understanding.*

(M7)

*You know, back in the day, the way they handled people with mental disorders was pretty standard. But I’ve seen a change in mental healthcare over the last couple of decades. They’re moving away from institutions and focusing more on treating people like, well, people! However, folks in the community still see mental health in that old way. That’s why it’s super important to have them visit our place, see what we’re up to now, and meet the people we’re helping. It’ll give them a chance to see things differently and understand these people in a new light.*

(M13)

#### 3.2.7. Support from Government Authority

The significance of government support was emphasized by numerous participants across different facilities. When communicating with residents is ineffective/unfeasible or residents are unwilling to compromise, the intervention of public authority emerges as the most effective approach.

*You know, when it’s a mix of public and private, no matter how much the residents resist, things seem to get sorted out without a hitch. Why? Because when the government steps in, it’s like a sure thing they’ll handle it. It’s like, as soon as the authorities genuinely get involved, the problem disappears like magic.*

(M5)

*If the government takes charge and the facility has solid governmental support, protest incidents become easier to resolve as some residents trust the government’s planning.*

(M8)

## 4. Discussion

The findings of this study indicate that the establishment of community mental health facilities in Taiwan faces significant challenges. First, the stigmatization and discrimination against people with mental disorders stem from societal learning. The adverse portrayal of people with mental disorders by the media significantly shapes the public’s perception of this demographic. Furthermore, it influences the preconceived notions regarding the potential impact of introducing community mental health facilities into the community. For instance, in both Taiwan and Hong Kong, there have been incidents involving people with mental disorders randomly committing acts of violence on the streets or within subway stations, shaping the portrayal of people with mental disorders as perpetrators of violence by the media [19]. Similarly, studies in the United States have revealed that community residents lack awareness about mental disorders, tend to lack empathy toward people with such conditions, and are often susceptible to influence from mainstream media [20]. Research conducted in New Zealand has revealed that societal discrimination against people with mental disorders persists, with community members perceiving a threat to their safety from people with such conditions [21]. Second, prevailing stereotypes about mental disorders in Taiwan hinder those people’s proactive seeking of services. Within the context of Asian societies, mental health issues are frequently attributed to individual deficiencies [22]. In Singapore, mental health challenges tend to be perceived as personal weaknesses [23]. In Taiwan, the lack of comprehensive understanding of mental health facilities, coupled with fear toward people with mental disorders, contributes to many Taiwanese people’s resistance to community mental health facilities [24]. In Macau, mental disorders are viewed as rare and incurable conditions, leading to fear and discrimination among the public [25]. In Japan, there is a relatively negative public perception of mental disorders, hindering the rights of people with mental disorders to seek medical treatment [26]. The study findings indicate that resistance against setting up community mental health facilities is associated with NIMBYism. Opponents believe that locating these facilities anywhere other than their “backyard” is preferable [27]. This situation is similar to that in Hong Kong, Canada, and the United States, where residents share similar concerns that establishing such facilities in their communities might threaten personal property security and negatively impact the community’s reputation [28].

Fourth, the findings of this study indicate that Taiwan’s community mental health facilities employ seven strategies to address community resistance, namely providing community services to foster amicable relations, promoting the establishment and sustenance of community friendships, and obtaining support from government authorities. New Zealand, the United States, and Canada emphasize community education to increase public awareness of mental disorders and reduce discrimination. New Zealand employs public education, such as mass media campaigns, to eliminate discrimination and fear toward people with mental disorders [29]. In the United States, local mental health facilities strive to alter/reshape public perceptions of mental disorders and utilize the mass media to communicate accurate information and care-related issues associated with mental disorders [30]. Similarly, Canada emphasizes strategies that involve publicity, public engagement, and fostering public understanding [31]. Government agencies develop methods for collecting public opinions on NIMBY issues and provide guidelines for addressing public opposition emotions [32,33]. In the Asian region, in Singapore, government agencies, healthcare providers, and community partners collaborate closely to reduce discrimination against people with mental disorders [34]. In Macau, there have been notable improvements in community mental health education in recent years. Government organizations have been actively involved in promoting mental health knowledge and reducing discrimination against people with mental disorders through annual Mental Health Day activities [35,36]. In South Korea, there is a strong emphasis on actively managing communities, thus reinforcing community residents’ understanding and attitudes toward people with mental disorders [37]. Furthermore, community surveys are conducted to understand community residents’ attitudes toward establishing community mental health facilities [37]. In Japan, the government has recently undertaken a series of initiatives to enhance community mental health care. These efforts aim to alter public attitudes toward mental disorders and restructure psychiatric mental health services, simultaneously reinforcing community support systems [38]. Fifth, governmental intervention represents one of the most efficacious methodologies in mitigating NIMBYism. Countries such as Singapore and the United States have adopted a legally oriented approach at the national level. This involves the implementation of clear legislative frameworks for land zoning and the enactment of legally binding strategies aimed specifically at the establishment of social welfare facilities. Such legal mechanisms can expedite the process of establishing community mental health facilities.

However, this study had several limitations. First, the study used convenience sampling, which limits the external validity of the study. Second, the participants in this study were exclusively healthcare professionals from community mental health facilities, and their viewpoints may not necessarily align with those of policymakers and expert scholars. Hence, future research should expand its scope to encompass policymakers and expert scholars, encouraging a three-way dialogue involving academia, government, and the industry. Third, the sample size was limited, and it suggests that further research explores the community protest status of different community mental health facilities. Fourth, this study relied on qualitative research methods to interview frontline facility healthcare professionals who have managed and handled community protests against the service agencies for people with mental illness in Taiwan. Subsequent research can explore the use of quantitative research surveys to collect feedback from the general public to provide an understanding of the public’s perceptions toward community mental health facilities.

## 5. Conclusions

The findings of this study reveal that community mental health facilities face stigmatization and discrimination from the local community regarding people with mental disorders. NIMBYism concerning these facilities is primarily associated with the users of these facilities being stigmatized due to their mental behaviors, leading to social exclusion by other community residents. The reasons behind the community residents’ resistance to community mental health facilities are varied and diverse, rooted in the public’s lack of understanding of mental disorders and entangled with numerous irrational beliefs. Furthermore, there exists a prevalent sense of fear towards people with mental disorders, reflecting a pervasive negative perception among the Taiwanese populace regarding these individuals. Community mental health facilities employ several strategies to address community resistance. These encompass providing community services to nurture amicable relationships, organizing or engaging in community outreach activities, delivering independent living training for people with mental disorders, nurturing local neighborhood connections, fostering the establishment and sustenance of community friendships, inviting residents to visit the facilities or introducing them to the community, and seeking government support. Today, people with mental disorders are entitled to equal rights to live within communities, and their integration into society has become a mainstream trend. However, social integration is a complex process. Therefore, it is essential to continuously explore and understand the interactions among service users of community mental health facilities, service providers, and other community residents.

## Figures and Tables

**Table 1:** Demographic characteristics of participants (*n* = 15).

Characteristics	Categories	N	%
Age (Years)	20–29	1	6.7
	30–39	6	40.0
	40–49	8	53.3
Gender	Male	7	46.7
	Female	8	53.3
Professional background	Social work	8	53.3
	Nursing	5	33.4
	Psychology	2	13.3
Job tenure (Experience)	1–3	1	6.7
	4–6	4	26.7
	7–9	10	66.6
Organizational attributes	Private	10	66.6
	Public	1	6.7
	government-owned and civilian-run	4	26.7
Geographical distribution	North	2	13.3
	Middle	4	26.7
	South	7	46.7
	East	2	13.3

**Table 2:** Summary of themes and subthemes emerging from the interviews.

Theme	Subtheme
1. Reasons behind community residents’ resistance against community mental health facilities	● 1.1 Diverse and varied negative perceptions
	● 1.2 Social stigmatization and exclusion, attributing unrelated community matters to people with mental disorders
	● 1.3 Fear of people with mental disorders: Reflection of social values
	● 1.4 Misunderstanding of people with mental disorders: Riddled with irrational Beliefs
2. Strategies employed by institutions in response to community residents’ resistance	● 2.1 Lack of clarification in organizational goals and role definition
	● 2.2 Organizing or engaging in community outreach activities
	● 2.3 Providing independent living training for people with mental disorders in the community
	● 2.4 Nurturing local neighborhood connections
	● 2.5 Facilitating the establishment and sustenance of community-based friendships
	● 2.6 Inviting residents to visit the community mental health facility or introducing the facility to the community
	● 2.7 Support from government authority

## Data Availability

The data presented in this study are available upon request from the corresponding author.

## References

[1] GBD 2019 Mental Disorders Collaborators (2022). Global, regional, and national burden of 12 mental disorders in 204 countries and territories, 1990–2019: A systematic analysis for the Global Burden of Disease Study 2019. Lancet Psychiatry..

[2] Moitra M., Owens S., Hailemariam M., Wilson K.S., Mensa-Kwao A., Gonese G., Kamamia C.K., White B., Young D.M., Collins P.Y. (2023). Global mental health: Where we are and where we are going. Curr. Psychiatry Rep..

[3] Ministry of Health and Welfare Taiwan. Mental Health Act. https://law.moj.gov.tw/ENG/LawClass/LawAll.aspx?pcode=L0020030.

[4] Executive Yuan, Taiwan A Stronger Social Safety Net for Taiwan. https://english.ey.gov.tw/News3/9E5540D592A5FECD/ce85ec40-08a7-4513-9c31-bc25ab77f15d.

[5] Duke J. (2010). Exploring Homeowner opposition to public housing developments. J. Sociol. Soc. Welf..

[6] Lee S.D. (2010). Perceptions and Attitude on Mental Health Service and People Living with Mental Illness among Community Leaders: A Focus in One Area. Master s Thesis.

[7] Esaiasson P. (2014). NIMBYism—A re-examination of the phenomenon. Soc. Sci. Res..

[8] Wood L., Birtel M., Alsawy S., Pyle M., Morrison A. (2014). Public perceptions of stigma towards people with schizophrenia, depression, and anxiety. Psychiatry Res..

[9] Angermeyer M.C., Matschinger H., Link B.G., Schomerus G. (2014). Public attitudes regarding individual and structural discrimination: Two sides of the same coin?. Soc. Sci. Med..

[10] Lasalvia A., Zoppei S., Bortel T.V., Bonetto C., Cristofalo D., Wahlbeck K., Vasseur S., Van Audenhove C., Weeghel J., Reneses B. (2013). Global pattern of experienced and anticipated discrimination reported by people with major depressive disorder: A cross-sectional survey. Lancet.

[11] Clement S., Williams P., Farrelly S., Hatch S.L., Schauman O., Jeffery D., Henderson R.C., Thornicroft G. (2015). Mental health-related discrimination as a predictor of low engagement with mental health services. Psychiatr. Serv..

[12] Yitbarek K., Birhanu Z., Tucho G.T., Anand S., Agenagnew L., Ahmed G., Getnet M., Tesfaye Y. (2021). Barriers and Facilitators for Implementing Mental Health Services into the Ethiopian Health Extension Program: A Qualitative Study. Risk Manag. Healthc. Policy.

[13] Patel V., Saxena S., Lund C., Thornicroft G., Baingana F., Bolton P., Chisholm D., Collins P.Y., Cooper J.L., Eaton J. (2018). The lancet commission on global mental health and sustainable development. Lancet.

[14] United Nationals Convention on the Rights of Persons with Disabilities. https://www.ohchr.org/en/instruments-mechanisms/instruments/convention-rights-persons-disabilities.

[15] Wolsink M. (1994). Entanglement of interests and mtives: Assumptions behind the NIMBY-theory on facility siting. Urban Studies.

[16] Wu Y.J., Cheng T.A., Minas H., Milton L. (2017). A history of mental healthcare in Taiwan. Mental Health in Asia and the Pacific: Historical and Comparative Perspectives.

[17] Graneheim U.H., Lundman B. (2004). Qualitative content analysis in nursing research: Concepts, procedures and measures to achieve trustworthiness. Nurse Educ. Today.

[18] Lincoln Y.S., Guba E.G. (1985). Naturalistic Inquiry.

[19] Legislative Council of Hong Kong Submission on Looking into Mental Health Services and Relevant Welfare Issues in Light of the MTR Arson Attack from a Deputation. Legislative Council of the Hong Kong Special Administrative Region—Panel on Welfare Services (Papers). https://www.legco.gov.hk/yr19-20/english/panels/ws/papers/ws_o3.htm.

[20] Schively C. (2007). Understanding the nimby and Lulu phenomena: Reassessing our knowledge base and informing future research. J. Plan. Lit..

[21] Star L., Mulgrew L., Akroyd S., Hemaloto S., Goodman K., Wyllie A. Research with Mental Health Service Providers-like Minds. https://www.likeminds.org.nz/assets/Uploads/research-with-mental-health-serviceproviders.pdf.

[22] Chiu M.Y.L., Chan K.K.L. (2007). Community attitudes towards discriminatory practice against people with severe mental illness in Hong Kong. Int. J. Soc. Psychiatry.

[23] Philomin L. Considerable Stigma against Mental Illness: Study. https://www.psychiatry.org/patients-families/stigma-and-discrimination.

[24] Yao C.T. (2015). A review of Taiwan’s community mental health policies: Current developments and future prospect. J. Nurs..

[25] Chi K.L. Is Mental Illness Terrible? Interview with the Director of Richmond Fellowship of Macau. https://aamacau.com/2013/12/10/%E7%B2%BE%E7%A5%9E%E7%97%85%E5%8F%AF%E6%80%95%E5%97%8E/.

[26] Setoya Y., Takeshima T. Japanese Mental Health System Reform Process and Comparisons with Australia. http://www.npo-jam.org/en/pdf/en_data_001.pdf.

[27] Tsang H.W.H., Tam P.K.C., Chan F., Cheung W.M. (2003). Stigmatizing attitudes towards individuals with mental illness in Hong Kong: Implications for their recovery. J. Community Psychol..

[28] Jiang S. (2013). How opposition protests affect government policy—Taking Macao as an example. Stud. Hong Kong Macao.

[29] Thornicroft C., Wyllie A., Thornicroft G., Mehta N. (2013). Impact of the “like Minds, like mine” anti-stigma and discrimination campaign in New Zealand on anticipated and experienced discrimination. Aust. N. Z. J. Psychiatr..

[30] Warner R. (2005). Local projects of the world psychiatric association programme to reduce stigma and discrimination. Psychiatr. Serv..

[31] Warner R. (2008). Implementing local projects to reduce the stigma of mental illness. Epidemiol. Psichiatr. Soc..

[32] Community Inclusion Awareness Committee A Toolkit for Opening Staffed COMMUNITY Homes in Manitoba. https://abilitiesmanitoba.org/wp-content/uploads/2019/01/Nimby-Toolkit-web.pdf.

[33] Habiter Montréal Strategy for the Inclusion of Affordable Housing -OCPM. https://ocpm.qc.ca/sites/ocpm.qc.ca/files/document_consultation/3aeng_0.pdf.

[34] Daud S. Nearly 10% of S’pore Residents will Suffer from Mental Health Issues, More Support to Reduce Social Stigma. Mothership.SG—News from Singapore, Asia and around the World. https://mothership.sg/2017/11/nearly-10-of-spore-residents-will-suffer-from-mental-health-issues-more-support-to-reduce-social-stigma.

[35] GIB Held a Series of Activities in “World Mental Health Day 2010”. https://www.who.int/campaigns/world-mental-health-day/2020/world-mental-health-day-campaign.

[36] Macau Daily Missionary Emphasis on Mental Health. http://www.macaodaily.com/html/2017-04/09/content_1170146.htm.

[37] Lee J.F., Lee Y.M., Lim K.Y., Lee H.Y. (1999). A survey for community attitudes towards the mentally-ill in Asian Area. J. Korean Neuropsychiatr. Assoc..

[38] Jung S., Kang B., Lee G. (2017). Changes in attitude towards people with mental illness in P-City, S. Korea: A comparison between the years 2000 and 2010. Asia Pac. J. Soc. Work Dev..

